# Population genetic structure of *Schistosoma bovis* and *S. curassoni* collected from cattle in Mali[Fn FN1]

**DOI:** 10.1051/parasite/2024035

**Published:** 2024-07-02

**Authors:** Assitan Diakité, Privat Agniwo, Abdoulaye Dabo, Bakary Sidibé, Boris A.E.S. Savassi, Ahristode Akplogan, Hassim Guindo, Laurent Dembélé, Moudachirou Ibikounlé, Safiatou Doumbo Niaré, Saidou Tembely, Jérôme Boissier

**Affiliations:** 1 Department of Epidemiology of Infectious Diseases, Faculty of Pharmacy, Malaria Research and Training Center (MRTC), University of Sciences, Techniques and Technologies of Bamako, Environnement, Santé, Sociétés (USTTB/UCAD/UGB/CNRST/CNRS) BP 1805, IRL3189 Bamako Mali; 2 Interactions Hôtes-Pathogènes-Environnements (IHPE), Univ. Montpellier, CNRS, Ifremer, Université de Perpignan Via Domitia 58 Avenue Paul Alduy Bâtiment R 66860 Perpignan France; 3 Centre de Recherche pour la lutte contre les Maladies Infectieuses Tropicales (CReMIT/TIDRC), Université d’Abomey-Calavi 01 BP 526 Abomey-Calavi Bénin; 4 Académie des Sciences du Mali, Baco-Djicoroni ACI Ouest Rue 619 Porte, 104 Bamako Mali

**Keywords:** *Schistosoma bovis*, *S. curassoni*, Slaughterhouse, Cattle, Genetic structure, Mali

## Abstract

Schistosomiasis is of medical and veterinary importance. Despite the critical situation of schistosomiasis in sub-Saharan Africa, few molecular epidemiological studies have been carried out to determine the role of animals in its transmission. In Mali, it has been over three decades since the last molecular study of animal schistosomes was carried out. It is now urgent to identify circulating strains of the parasite because of potential interactions with other schistosome species, which could complicate disease control. The aim of our work was to study the composition and genetic structure of schistosome populations collected from cattle. The prevalence of schistosome was 23.9%, with the prevalences of *Schistosoma bovis (Sb)* and *S. curassoni (Sc)* estimated at 12.6% and 9.8%, respectively. No hybrid strains or *S. haematobium* were found. The parasites displayed distinct geographical distribution with *Sb* dominant in Bamako (78.8% and 98% in Central Bamako Slaughterhouse and Sabalibougou Slaughterhouses, respectively) and *Sc* dominant in Kayes (95.3%). Of the 476 parasites with a complete genetic profile, 60.4% were pure *Sc*, and were mainly from Kayes. We identified two clusters at the site level (Fst of 0.057 and 0.042 for *Sb* and *Sc*, respectively). Cluster 1 was predominantly composed of pure *Sb* parasites and cluster 2 was mainly composed of pure *Sc* parasites, from Bamako and Kayes, respectively. Our study shows that cattle schistosomiasis remains endemic in Mali with *S. bovis* and *S. curassoni*. A robust genetic structure between the different schistosome populations was identified, which included two clusters based on the geographical distribution of the parasites.

## Abstract (fr)

La schistosomiase revêt une grande importance médicale et vétérinaire. Malgré la situation critique de la schistosomiase en Afrique subsaharienne, peu d’études épidémiologiques moléculaires ont été réalisées pour déterminer le rôle des animaux dans sa transmission. Au Mali, cela fait plus de trois décennies que la dernière étude moléculaire des schistosomes animaux a été réalisée. Il est désormais urgent d’identifier les souches circulantes du parasite en raison des interactions potentielles avec d’autres espèces de schistosomes, ce qui pourrait compliquer la lutte contre la maladie. Le but de notre travail était d’étudier la composition et la structure génétique des populations de schistosomes collectées chez des bovins. La prévalence des schistosomes était de 23,9 %, celles de *Schistosoma bovis* (*Sb*) et de *S. curassoni* (*Sc*) étant respectivement estimées à 12,6 % et 9,8 %. Aucune souche hybride ni *S. haematobium* n’ont été trouvés. Les parasites présentaient une répartition géographique distincte avec *Sb* dominant à Bamako (respectivement 78,8 % et 98 % aux Abattoirs Centraux de Bamako et aux Abattoirs de Sabalibougou) et *Sc* dominant à Kayes (95,3 %). Sur les 476 parasites ayant un profil génétique complet, 60,4 % étaient des *Sc* purs, et provenaient principalement de Kayes. Nous avons identifié deux clusters au niveau du site (Fst de 0,057 et 0,042 pour *Sb* et *Sc*, respectivement). Le groupe 1 était principalement composé de parasites *Sb* purs et le groupe 2 était principalement composé de parasites *Sc* purs, provenant respectivement de Bamako et de Kayes. Notre étude montre que la schistosomiase bovine reste endémique au Mali, avec *S. bovis* and *S. curassoni*. Une structure génétique robuste entre les différentes populations de schistosomes a été identifiée, comprenant deux groupes basés sur la répartition géographique des parasites.

## Introduction

Schistosomiasis continues to be a major public health threat in tropical and subtropical areas. This parasitosis belongs to a group of Neglected Tropical Diseases which collectively affect 1 billion people worldwide, and of which schistosomiasis accounts for infections in 250 million people in 78 countries [5]. It was estimated that worldwide, approximately 165 million animals are also infected with the parasite, which causes hemorrhagic enteritis, anemia, cachexia and for most, death [10]. In Mali, compared to the numerous studies done on human schistosomiasis, livestock schistosomiasis has received little attention and the most recent studies were carried out more than 30 years ago, hence the need to update existing data [36]. Of the 23 species of *Schistosoma* known worldwide, only nine have been identified to be of significant veterinary importance, especially for ruminants in Asia and Africa; these include: *Schistosoma mattheei; S. bovis* (*Sb*)*; S. curassoni* (*Sc*)*; S. spindale; S. indicum; S. nasale; S. incognitum; S. margrebowiei; and S. japonicum* [4, 22, 35]. Of these, two species, *Sb* and *Sc* are native to West Africa, six are widely distributed in Central and Eastern Africa [22, 26, 28, 31, 36, 41, 42] and one, *S. japonicum* is native to Asia [40]. The prevalence of *Sb* and *Sc* has been reported in some countries [26, 28, 36, 41, 42]. A study by Rollinson [36] in 1990 reported a high prevalence of schistosomiasis in cattle across three West African countries, namely Gambia (*S. bovis*: 14.3–27%), Senegal (*Sb* plus *Sc* and hybrids: 0–41.3%) and Mali *(Sb*, *Sc* and hybrids) with 62.8% in Bamako and 85.1% in Mopti. The two most recent molecular epidemiology studies of animal schistosomes were carried out in Senegal and Cameroon [12, 21]. In Cameroon, a recent study showed a 19.5% prevalence of adult schistosome worms in cattle [12], and prevalences of 92% and 8% were reported for *S. bovis* and *S. curassoni* in cattle in Senegal, respectively [21]. The control or elimination of cattle schistosomiasis will contribute to a substantial increase in the weight of the animals, thus improving animal productivity, which in turn will increase the incomes of livestock farmers and the availability of quality meat for consumption.

The schistosome parasite has a complex life cycle. Individuals are dioecious rather than hermaphroditic, as is the case with most other trematodes like liver flukes or tapeworms. Gonochorism allows interspecific interactions between males and females within their definitive hosts. Today, parasite hybridization is an emerging public health problem, as the cohabitation of human populations and domestic or wildlife animals. Increased mobility of populations creates opportunity for new agents to combine, and concerning parasites, this includes new interactions between parasites of different lineages or species within individual hosts [20, 29, 39]. The genetic variation of schistosome species and populations over time and space is based on the hybridization phenomenon and can only be described through molecular epidemiological studies. While many studies on population genetics of schistosomes in humans have been conducted [35], only few studies exist for animal populations. Parasite hybridization is a biological phenomenon that is gaining renewed interest as new molecular tools have enabled hybridization to be more easily detected, and because the repercussions in the evolution of infections are still largely unknown [19]. Hybridization is known in some cases to increase the virulence of the parasite towards its vertebrate hosts and to allow the parasite to broaden its spectrum of intermediate and/or definitive hosts [19, 47]. The first cases of hybridization between schistosomes, notably between *Sb* and *Sc* in animals, were reported in Africa (Mali & Senegal) by Rollinson [36]. In Senegal, a recent study found a high occurrence of bidirectional hybridization between these animal schistosome species, and this is the second piece of conclusive evidence of natural hybridization between *Sb* and *Sc* [44]. Also in the Niger River valley region, transmission of human and bovine schistosomiasis has been identified, but it seems that the transmission of the different species was limited [32].

The only population genetic study carried out on livestock schistosome was done in Cameroon [12]. The present study aims to understand the genetic composition and the population genetic structure of schistosomes collected from cattle in Mali. Specifically, we proposed (i) to determine the prevalence of schistosomes in cattle in Kayes and Bamako slaughterhouses; (ii) to identify the schistosome species and the possible presence of *S. haematobium* group hybrid parasites using genetic markers (ITS, 18S and Cox1); and (iii) to describe the genetic structure of schistosomes in cattle based on microsatellite data.

## Material and methods

### Study sites

**Figure 1:**
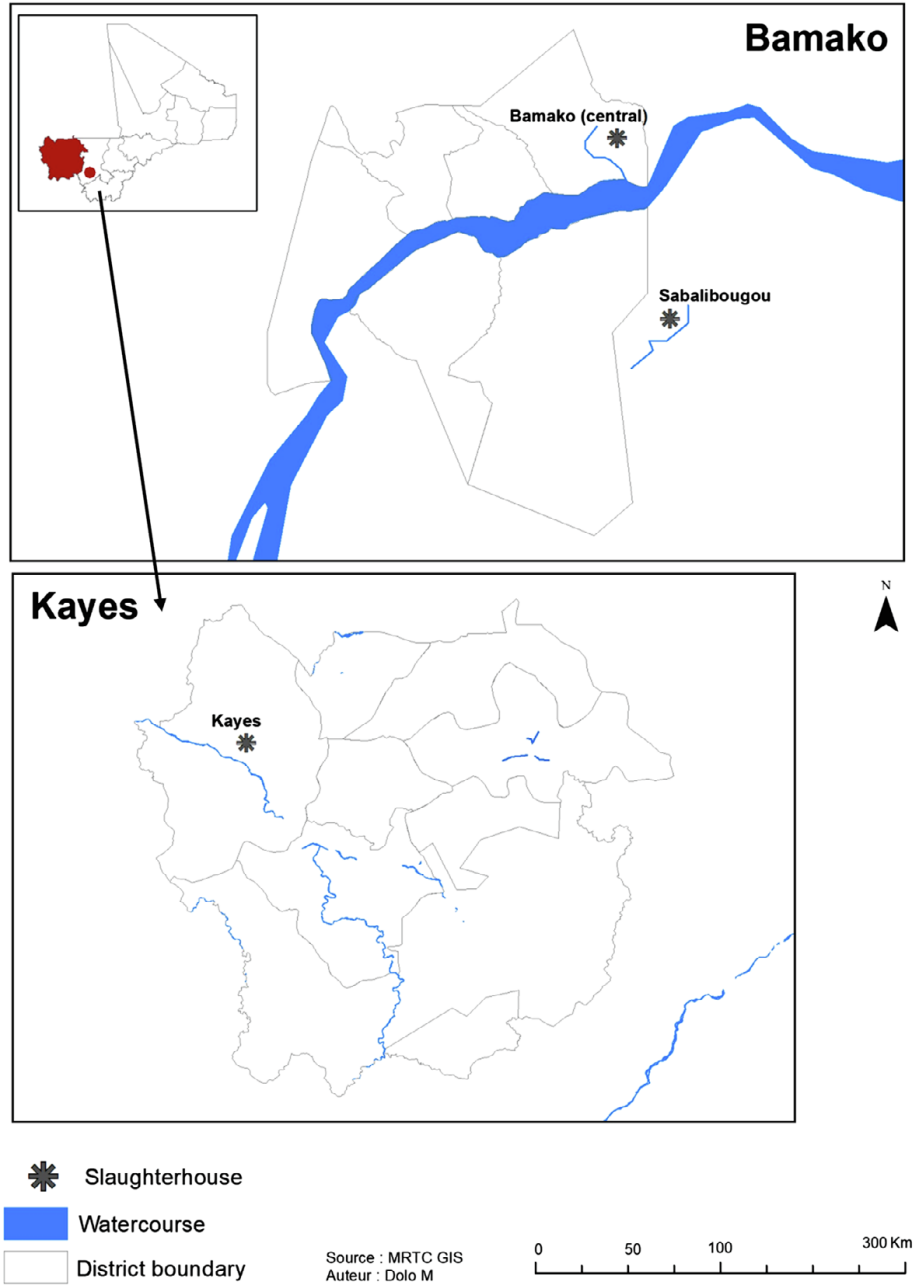
Map of Mali showing selected slaughterhouses.

Mali ranks first in the West African Economic and Monetary Union (UEMOA) and second in the Economic Community of West African States (ECOWAS) zone in terms of bovine breeding, with an estimated 12,474,462 heads of cattle in its national herd [34]. A cross-sectional study was performed for two collections, in September (end of the rainy season) and December (cold dry season) 2021 in two areas: at the Central Bamako Slaughterhouse (CBS) and Sabalibougou Slaughterhouses (SS) in District of Bamako (Koulikoro region) and at the Kayes Slaughterhouse (KS) in Kayes region (Fig. 1). Unlike the two slaughterhouses located in the District of Bamako, where animals from all regions of the country converge, Kayes is an extensive livestock farming area. The two areas were specifically selected because they are known to be endemic for *S. haematobium (Sh)* [1], a human schistosome species. *Sb* and *Sc* are two schistosome species that infect animals [36]. In Mali, the Ministry of Livestock and Fisheries, through the National Veterinary Services Department, is the official body responsible for animal health. Anthrax, contagious bovine pneumonia, and plague of small ruminants are among the diseases managed by this service. No specific treatment is currently provided for schistosomiasis, even on small-scale dairy farms. Parasites were isolated and conditioned at the Department of Epidemiology and Parasitic Diseases (DEAP) of the Malaria Research and Training Center (MRTC) in Mali, and molecular manipulations were carried out at the Host-Pathogen-Environment Interactions (IHPE) laboratory in France.

### Parasite collection

Collections of adult schistosomes were made between 0:00 am and 3:00 am from slaughtered cattle. Each collection session was followed by the identification of the animal from which the worms were collected, on a pre-established survey form. The site, the season, the number of worms recovered from each animal and the organ from which the worm was collected were recorded. In each slaughterhouse, intestines and ceca were randomly collected from slaughtered animals. The mesenteric veins surrounding the small intestines, and the veins which irrigate the ceca were carefully observed macroscopically by transparency for the presence of schistosome worms (whitish-colored worms through the vein). The worms were removed from the veins using forceps and placed in a 15 mL falcon tube containing physiological water, labelled with the bovine identifier and the removed organ. Once in the laboratory, the worms were isolated one by one using fine tweezers and stored individually in pure ethanol.

### Extraction of genomic DNA (gDNA)

To extract genomic DNA (gDNA), the ethanol was first removed using a pipette, and then the tube containing the parasite was kept open and dried using the Speed Vac set at 14,000 rpm. Genomic DNA of 491 parasites was extracted using a DNeasy blood and tissue kit (QIAGEN, Hilden, Germany), following the manufacturer’s protocol, and the DNA was eluted in a total of 100 μL.

### Rapid diagnostic polymerase chain reaction (RD-PCR) for Cox1 and tetraprimer amplification refractory mutations system – Polymerase chain reaction (T-ARMS-PCR) for ITS2 and 18S genotyping

Identification of Cox 1 profiles from 491 worms was done by Cox1 rapid diagnostic multiplex PCR (RD-PCR) with primers targeting two species groups: *Sh* and *Sb* or *Sc* [43]. In short, we used a universal reverse (Shmb.R) and two forward primers specific to three schistosome species: *Sh*F (120 bp), *Sb*F (260 bp – this band is the same for both *Sc* and *Sb*). Each PCR was carried out in a total volume of 10 μL containing 4 μL of ultra-pure water, 2 μL of buffer (Green GoTaq Flexi buffer, 5×; Promega; Madison, WI, USA), 0.6 μL of MgCl_2_ (Promega) at 25 mM, 0.2 μL of mixture of dNTPs (Promega) at 10 mM; 1 μL of 10× primer mix with 4 μL of each armor in 84 μL of ultra-pure water), 0.2 μL of GoTaq^®^ Polymerase G2 Hot Start at 5 U (Promega), and 2 μL of DNA extract. After 45 cycles of amplification, the PCR products (Cox1) were visualized on a 2% agarose gel at 135 V for 40 min using the 100 bp DNA size marker (Promega) to estimate the size of the bands. We used the T-ARMS-PCR method developed in a recent study [32] to detect parasite nuclear genotypes (ITS/18S). Each partial PCR amplification of nuclear DNA was performed in a total volume of 25 μL, containing 3 μL of DNA template, 5 μL of GoTaq^®^ Flexy buffer (Promega), 1, 5 μL of 25 mM MgCl_2_, 0.5 μL of a mixture of 8 prediluted primers (12 μM external primers and 15 μM for internal ones), 0.2 μL of dNTP solutions at 10 mM each, 0.5 μL of GoTaq^®^ Taq polymerase G2 Hot Start (Promega) and 10.75 μL of milli-Q water. After 28 cycles of amplification, PCR products were examined on 1.8% agarose gels at 135 V for 40 min using the 100 bp DNA size marker (Promega) for size estimation. Four to six bands could be observed on the agarose gels, which allowed for the differentiation between *Sc, Sb, Sh* and all hybrid combinations [4].

### Sanger sequencing

***Cox1***: Because the RD-PCR does not differentiate between *Sc* and *Sb,* we sequenced Cox 1 partial region (≈918 bp) for 188 parasites using a previously published protocol [23]. We considered the T-ARMS-PCR results as an indicator of sample selection for sequencing. We sequenced 107 and 81 parasites with either the *Sb* and *Sc* T-ARMS-PCR profile, respectively. The size fragment was checked on 5% agarose gel and PCR products were sent without purification to a subcontractor for sequencing (Genoscreen, Lille, France). Cox1 sequences were then compared to a reference sequence from GenBank (Accession number: AJ519521.1 and MT579424.1 for *Sb* and *Sc*, respectively).

***18S***: In order to double check several of the T-ARMS-PCR results, partial 18S regions (329 bp) were amplified and sequenced on 26 and 20, pure *Sb* and pure *Sc* profiles, as previously described [32]. The size fragment was checked on 5% agarose gel and PCR products were sent without purification to a subcontractor for sequencing (Genoscreen). The sequences obtained were compared with reference sequences (Accession number: AY157238.1 and AY157236.1 for *Sb* and *Sc*, respectively). At position 297, a C base identified *Sb* and a T base identified *Sc* [4].

### Microsatellite genotyping

Microsatellite genotyping was performed on parasites collected from the three slaughterhouses. Only samples of parasites with a well-defined profile for each of the three genes (Cox1, ITS and 18S) were selected for microsatellite genotyping. The worms were individually genotyped using the PCR multiplex kit from QIAGEN and two panels of 8 loci: (*Sh*9, *Sh*3, C102, *Sh*1, *Sh*14, *Sh*6, C111, and *Sh*7) & (*Sh*13, *Sh*4, *Sh*11, *Sh*15, *Sh*2, *Sh*5, *Sh*10, and *Sh*12) [45]. The PCR mixture consisted of 5 μL of QIAGEN MM 2×, 1 μL of 10× microsatellite primer mix and 4 μL of DNA extract for a final volume of 10 μL. Thermal cycling was performed in a PerkinElmer 9600 Thermal Cycler (PerkinElmer, Waltham, MA, USA): pre-denaturation at 95 °C for 15 min followed by 40 cycles. Microsatellite PCR products were sent to Genoscreen for genotyping. After verification and correction using GeneMarker software, the data were then transferred to Excel to determine the position of the various loci.

### Statistical analyses

Data were entered using Excel software. Calculations of prevalence of infection were performed using SPSS v 23.0 (IBM) software. Logistic regressions were used to infer the relationship between variables (slaughterhouse, season, and organ of sampling) and genetic profile. Lastly, *p*-values less than 0.05 were considered significant.

### Genetic data analysis

Observed (Ho) and expected (He) heterozygosity, number of alleles (A), allelic richness (Ar), and inbreeding coefficient (Fis) at each microsatellite locus and for each population were calculated using FSTAT 2 software, 9.3.2 [16]. Population refers to the set of parasites collected from a given slaughterhouse (CBS, SS or KS). These parameters were compared according to the different populations or species using either Wilcoxon or Friedman pairwise rank tests followed by Nemenyi *post-hoc* tests.

First, population genetic structure was assessed using Fst indices [46]. Second, Genetix software [3] enabled us to visualize the potential genetic structure between individuals according to species using principal component analysis (PCA). Third, we evaluated the probability that the microsatellite database followed a defined number of clusters from *K* = 1 to *K* = 4 using Structure software [33]. We considered 10 runs for each trial, each of which consisted of a running period of 50,000 following by 250,000 MCMC iterations. The Δ*K* values were then calculated in *R* to determine the most likely number of clusters [13]. Finally, 10 additional runs were calculated for the best *K* using a running period of 50,000 and 500,000 iterations. The probability of each worm belonging to each cluster was averaged over the 10 runs and graphically represented using CLUMPP, version 1.1.2 [14] and DISTRUCT, version 1.1 [37].

## Results

### Schistosome prevalence in cattle

Animals slaughtered in slaughterhouses were mainly local Zebu breeds (*Bos taurus indicus*) and less commonly crossbreeds with N’dama (*B. taurus taurus*). Post-mortem examination of veins surrounding the small intestine and the cecum of cattle from Bamako and Kayes slaughterhouses revealed the presence of adult schistosome worms. Out of 398 cattle examined at the three slaughterhouses (two in Bamako and one in Kayes), 23.9% (95/398) were found to be infected by schistosomes (Table 1). Of these 95 cattle, we obtained the species of parasite involved for 89 individuals, with 50 animals infected by *Sb* parasites and 39 animals infected by *Sc* parasites. We can therefore estimate prevalences of 12.6% (50/398) and 9.8% (39/398) for *Sb* and *Sc*, respectively. Of note, 4/89 were co-infected by both species (1 from CBS and 3 from KS). The highest prevalence was recorded in KS (44.6%) compared to CBS (18.4%). More worms were recovered from the intestine (22.9%) compared to cecum veins (5%). No statistical difference in the prevalence was observed between seasons (*p =* 0.12). Sixteen bovines (6.5%) had parasites in both the small intestine and the cecum.

**Table 1:** Number (%) of worms recovered in cattle in three transmission sites in Mali according to the season of collection and their localization (veins surrounding the small intestine or the cecum).

		Cattle examined	Cattle infected	*p*-value	Worms collected in veins surrounding small intestine	*p*-value	Worms collected in veins surrounding the caecum	*p*-value
Area								
Bamako	CBS	140	31 (22.1)	*p* < 0.0001	28 (20.0)	*p* < 0.0001	3 (2.1)	*p* < 0.0001
	SS	175	27 (15.4)		27 (15.4)		0 (0)	
Total		315	58 (18.4)		55 (17.5)		3 (0.9)	
Kayes	KS	83	37 (44.6)		36 (43.4)		17 (20.5)	
Total		83	37 (44.6)		36 (43.4)		17 (20.5)	
Season								
	Rainy	268	69 (25.7)	*p = 0.12*	65 (24.3)	*p* = 0.2	20 (7.5)	*p* < 0.0001
	Dry	130	26 (20.0)		26 (20.0)		0 (0)	
Total		398	95 (23.9)		91 (22.9)		20 (5.0)	

CBS; Central Bamako Slaughterhouse; SS: Sabalibougou Slaughterhouses; KS: Kayes Slaughterhouse.

### Schistosome species identification

Of the 616 adult worms collected, 491 were analyzed genetically. Correct genetic profiles for 491 and 476 worms were obtained using RD-PCR and T-ARMS-PCR, respectively. Results from these analyses indicated that the majority of the parasites originated from KS 58.4% (278/476) (Table 2). The T-ARMS-PCR revealed either *Sc* or *Sb* homozygous profiles and no hybrids were identified (Table 2). All 18S sequences (26 and 20 with *Sb* and *Sc* T-ARMS-PCR profile, respectively) confirmed the T-ARMS-PCR profiles. The RD-PCR revealed only the *Sb/Sc* profile and not the *Sh* profile. All 107 parasites with a homozygous *Sb* T-ARMS-PCR profile showed a typical Cox1 *Sb* sequence and all 81 parasites with a homozygous *Sc* T-ARMS-PCR profile showed a typical Cox1 *Sc* sequence. Because we randomly selected 40% of the parasites for sequencing, we assumed that the T-ARMS-PCR (*SbxSb* or *ScxSc*) profile reflected the Cox1 profile (*Sb* or *Sc*). *Sb_SbxSb* profiles were predominantly present in Bamako with 78.8% in CBS and 98% in SS, respectively. All haplotypes are available on GenBank with access numbers for Cox1: *Sb* (PP654220 to PP654290); *Sc* (PP654291 to PP654330) and 18S: *Sb* (PP654446, PP654447, PP654448); *Sc* (PP654443, PP654444, PP654445).

**Table 2:** Multivariate analyses of adult worm species according to the site and the organ.

Regions			Total	*Sb_SbxSb*	*Sc_ScxSc*	*p*
Bamako	CBS	Small Intestine	95	75 (78.9)	20 (21.1)	*p* < 0.0001
		Cecum	4	3 (75)	1 (25)	
		Total	99	78 (78.8)	21 (21.2)	
	SS	Small Intestine	99	97 (98)	2 (2)	*p* < 0.5
		Cecum	0	0	0	
		Total	99	97 (98.0)	2 (2.0)	
Kayes	KS	Small Intestine	196	10 (5.1)	186 (94.9)	*p* < 0.0001
		Cecum	82	3 (3.7)	79 (96.3)	
		Total	278	13 (4.7)	265 (95.3)	
	Total		476	188 (39.5)	288 (60.5)	

CBS; Central Bamako Slaughterhouse; SS: Sabalibougou Slaughterhouses; KS: Kayes Slaughterhouse.

### Tropism of schistosome species

The vast majority of worms analyzed (81.9%) were localized in the veins surrounding the small intestine (Table 2). The small number of *Sc_ScxSc* parasites collected from Bamako were also principally localized in the veins surrounding the small intestine. In contrast, most parasites from KS exhibited an *Sc_ScxSc* profile (95.3%). The localization of these worms is more shared, with 82% (390/476) localized in the veins surrounding the small intestine.

### Microsatellite analysis

#### Genetic diversity

Among the 476 parasites with complete mitochondrial and nuclear profiles, 166 were genotyped using 16 microsatellite markers. Parasite genetic diversity indices (He, Ho, A, Ar, and Fis) at each marker and for each slaughterhouse are presented in Table 3. The Friedman test showed a difference between populations for all calculated indices, He (*χ*^2^ = 8, df = 2, *p* = 0.011), Ar (*χ*^2^ = 8.375, df = 2, *p* = 0.015) and Fis (*χ*^2^ = 12.5, df = 3, *p* = 0.0017) (Table 3). The Nemenyi *post-hoc* tests revealed that the difference in Ar and He observed was significant between CBS and KS (*p* = 0.013 and *p* = 0.009, respectively). For Fis, a significant difference was observed between SS and KS (*p* = 0.002). Table 4 represents the overall gene diversity (He) and allelic richness (Ar) for *Sb_SbxSb* and *Sc_ScxSc*: mean He and Ar are both higher for *Sb_SbxSb* than for *Sc_ScxSc,* but the difference is only significant for He (*W* = 116, *p* = 0.21 for Ar, *W* = 116, *p* = 0.01 for He) (Table 4).

**Table 3:** Genetic diversity indices of schistosome worms collected in cattle in three Malian slaughterhouses. Expected (He) and observed (Ho) heterozygosity, total number of alleles (A), allelic richness (Ar), and inbreeding coefficient (Fis) for each microsatellite locus and for each sampled site in Mali.

Slaughterhouse	Sh9	Sh3	C102	Sh1	Sh14	Sh6	C111	Sh7	Sh13	Sh4	Sh11	Sh15	Sh2	Sh5	Sh10	Sh12	Moy	Std
CBS (*n* = 55)																		
He exp	0.84	0.89	0.89	0.90	0.65	0.76	0.71	0.91	0.92	0.89	0.62	0.40	0.95	0.72	0.83	0.64	0.78	0.12
He Obs	0.40	0.71	0.24	0.67	0.67	0.33	0.44	0.66	0.90	0.87	0.48	0.32	0.96	0.29	0.71	0.58	0.57	0.22
A	13	16	13	13	8	8	8	20	17	18	10	7	22	7	12	10	12.63	3.88
Ar	10.83	13.94	12.42	11.84	6.51	7.06	6.99	16.11	15.64	14.87	8.37	5.74	19.55	6.83	10.55	7.69	10.93	3.48
Fis	0.51	0.20	0.73	0.25	−0.03	0.56	0.38	0.28	0.02	0.01	0.22	0.19	−0.02	0.60	0.14	0.09	0.26	0.19
SS (*n* = 42)																		
He exp	0.75	0.87	0.88	0.90	0.67	0.76	0.67	0.88	0.93	0.89	0.52	0.23	0.93	0.68	0.76	0.58	0.74	0.14
He Obs	0.46	0.82	0.30	0.89	0.59	0.40	0.54	0.54	0.91	0.88	0.50	0.21	0.94	0.31	0.83	0.63	0.60	0.24
A	9	17	14	12	5	7	7	12	17	15	4	3	16	6	8	3	9.69	4.40
Ar	8.02	15.25	14.00	11.73	4.99	6.80	6.49	11.49	16.49	14.09	3.91	2.69	15.67	5.83	7.87	3.00	9.27	4.23
Fis	0.38	0.06	0.69	0.01	0.12	0.47	0.19	0.39	0.02	0.01	0.05	0.08	−0.02	0.54	−0.11	−0.09	0.17	0.20
KS (*n* = 69)																		
He exp	0.56	0.83	0.69	0.16	0.36	0.27	0.22	0.21	0.94	0.91	0.81	0.19	0.82	0.28	0.68	0.83	0.55	0.27
He Obs	0.10	0.60	0.03	0.06	0.3	0.07	0.2	0.08	0.9	0.9	0.5	0.13	0.8	0.09	0.72	0.90	0.53	0.30
A	6	19	11	7	4	6	5	5	21	15	18	5	21	5	8	10	10.38	5.34
Ar	4.69	14.87	10.33	4.42	3.41	5.20	4.01	4.45	18.14	12.83	14.15	4.17	16.08	4.59	7.33	8.79	8.59	4.38
Fis	0.79	0.29	0.96	0.62	0.21	0.73	0.06	0.65	0.06	−0.02	0.33	0.27	0.01	0.69	−0.04	−0.09	0.34	0.30
Total (*n* = 166)																		
A	15	25	19	14	8	10	10	22	24	20	18	7	26	8	13	11	15.63	5.58
Ar	8.66	15.29	14.02	11.00	5.26	7.05	5.96	13.08	17.39	13.70	10.79	4.36	19.61	7.02	10.50	7.95	10.73	3.63

**Table 4:** Allelic richness and genetic diversity for *Sc_ScxSc* and *Sb_SbxSb* genotypes.

	Allelic richness (Ar)		Gene diversity (He)	
	*Sc_ScxSc*	*Sb_SbxSb*	*Sc_ScxSc*	*Sb_SbxSb*
Sh9	2.86	12.06	50%	81%
Sh3	16.71	16.8	79%	88%
C102	10	16.74	62%	88%
Sh1	1	13.09	–	90%
Sh14	2.85	6.5	25%	70%
Sh6	5.18	7.91	27%	76%
C111	4.42	7.59	21%	69%
Sh13	20.92	15.71	94%	92%
Sh4	14.28	15.39	90%	87%
Sh11	16.5	3.99	82%	49%
Sh15	5.78	4	25%	29%
Sh2	17.91	17.38	81%	93%
Sh5	4.76	6.4	28%	74%
Sh12	9.64	4.75	81%	57%
Mean	9.49	10.59	57%	75%
SE	1.76	1.39	8%	5%

#### Population genetic structure

**Figure 2:**
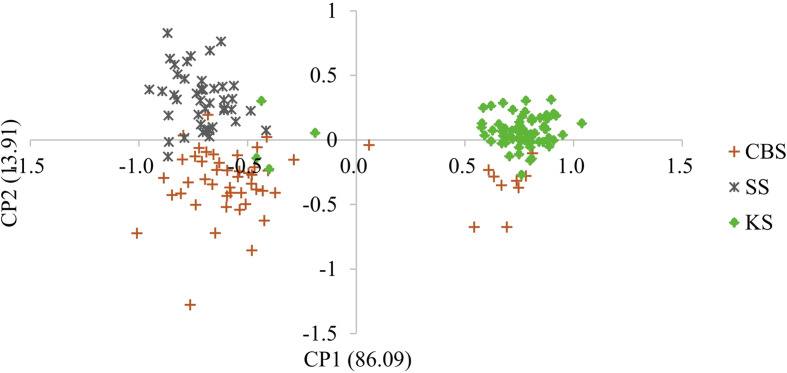
Principal component analysis (PCA) of schistosome worm parasite recovered from three slaughterhouses in Mali. Each point represents a parasite, and the color label corresponds to the population of origin (CSB, SS or KS). The first and second axes explain 86.09% and 13.91% of the genetic variation, respectively.

**Figure 3:**
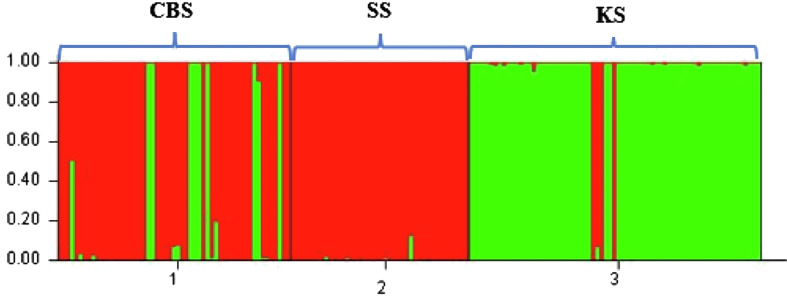
Bar plot showing the population genetic structure of 491 schistosome worms collected in three slaughterhouses in Mali, using Structure software. Each column represents one worm. The colors show the proportion of contribution of each cluster to each genotype. The cluster structure is for *K* = 2. The geographic populations are CBS (1), SS (2) and KS (3).

Whether we consider the PCA (Fig. 2) or Bayesian approach (Fig. 3), two groups were clearly identified. The first group is mainly composed of parasites from Bamako (CBS or CS) and the second group is mainly composed of parasites from Kayes (KS). According to the parasite genotyping, this structure is more a result of the differences between species than the sampling site. The few parasites from the Kayes group that clustered with the Bamako group are *Sb_SbxSb*, and conversely the few parasites from the Bamako group that clustered with the Kayes group are *Sc_ScxSc* parasites. The PCA first axes cluster the genotypes, and the second axes cluster the sites. The Bayesian analysis (Fig. 3) also shows that the vast majority of parasites are either assigned to cluster 1 and possess a *Sb_SbxSb* profile or to cluster 2 and possess a *Sc_ScxSc* profile. A single parasite from Bamako shows a 50/50 assignment probability between cluster 1 and 2. This parasite has an *Sb_SbxSb* profile in both PCR and sequencing and thus has not been identified as a hybrid.

**Figure 4:**
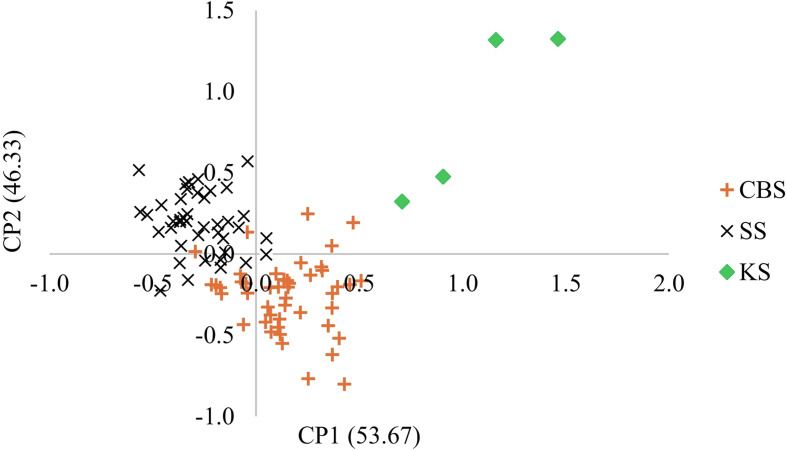
Principal component analysis (PCA) of *Schistosoma bovis* worms recovered from three slaughterhouses in Mali. Each point represents a parasite, and the color corresponds to the population of origin (CSB, SS or KS). The first and second axes explain 53.67% and 46.33% of the genetic variation, respectively.

**Figure 5:**
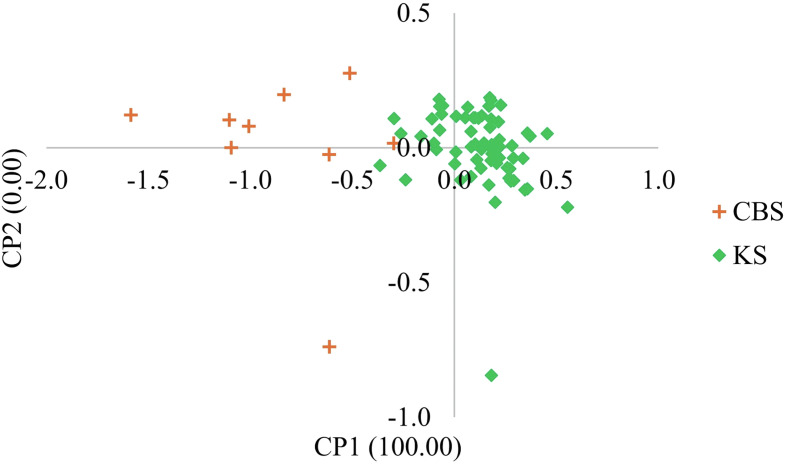
Principal component analysis (PCA) of *Schistosoma curassoni* worms recovered from three slaughterhouses in Mali. Each point represents a parasite, and the color corresponds to the population of origin (CSB, SS or KS). The first and second axes explain 100.00% and 0.00% of the genetic variation, respectively.

PCAs by species showed that regardless of the species, *Sb* (Fig. 4) or *Sc* (Fig. 5), the parasites are structured according to the collection site. Pairwise Fst value between *Sb_SbxSb* and *Sc_ScxSc* genotypes is 23%. Table 5 presents the Fst values between genotypes at different sites. Except for the *Sb_SbxSb* genotype between SS and CBS, the Fst values range between 4.2% and 5.7%.

**Table 5:** Pairwise estimates of Fst values for microsatellite DNA genotype (below the diagonal) of *Sb* and *Sc* parasite populations as a function of slaughterhouse.

*Sb_SbxSb*	CBS	SS
SS	0.0043	–
KS	0.044	0.057
*Sc_ScxSc*		
KS	0.042	

CBS; Central Bamako Slaughterhouse; SS: Sabalibougou Slaughterhouses; KS: Kayes Slaughterhouse.

## Discussion

Our study focused on the epidemiology, molecular characterization, and population genetics of *Schistosoma* spp. that infect cattle in Mali. The study was conducted in abattoirs in two regions, the CBS and the SS in the District of Bamako and the KS. The results showed that the livestock schistosomiasis was endemic in the two regions with a global prevalence of 23.9%. The highest rate was observed in KS (44.6%) compared to CBS and SS (18.4%). The prevalence of *Sc* in our study was estimated at 9.8%. This prevalence is similar to that reported in Cameroon (10.03%) in domestic ruminants, using morphological egg criteria [30], but lower than that observed in Bamako (Mali) for cattle (30.8%) using an iso-enzymatic method [36]. Using a molecular approach, *Sc* was also reported in Barkedji and Linguère in Senegal, with an estimated prevalence of 73% in sheep, 84% in goats, and 8% in cattle [21]. The low prevalence of *Sc* in our study may be due to the final host examined (cattle). As previously observed in Senegal [21], *Sc* preferred goats and sheep as hosts. The prevalence of *Sb* in our study was estimated at 12.6%. The prevalence of *Sb* varies greatly from one study to another according to the host species concerned. In sheep, prevalence rates ranging from 4% to 20% have been observed in Senegal [21, 27]. In goats, a prevalence of 15% has been observed in Senegal [21]. Prevalence rates are usually higher in cattle, with 19.5% in Cameroon [12] and 92% in Senegal [21]. The presence of schistosomes is dependent on the presence of intermediate snail hosts. Unfortunately, too few studies exist on the distribution of snails in Africa, particularly those that transmit schistosomes that infect animals. In West African countries, the snail species that hosts *Sb* may be *Bulinus truncatus, B. forskalii* or *B. globosus* [25], whereas *B. umbilicatus* is described as the main host of *Sc* [11]. In Mali, no study has yet looked specifically at snails as intermediate hosts of *Sc* or *Sb*, even though various intermediate hosts of schistosomiasis have been documented (*B. truncatus, B. globosus, B. umbilicatus, B. forskalii*, *and B. senegalensis*) [9, 24]. The snail *B. umbilicatus* has only been identified in the Mopti region, but the authors of the study were unable to determine the species of schistosome it transmitted [24]. Despite the specific distribution of snails due to ecological factors, animals slaughtered in Kayes or Bamako can be moved over great distances (600 km separate the livestock supply areas of Mopti from Bamako and those of Kayes and Nara on the Mauritanian border), which can bring them into contact with different transmission sites. No significant variation in prevalence was found with respect to season. The seasonal movement of animals over large distances to find new pastures and water points (transhumance) in Mali promotes their exposure to a variety of infected water bodies.

During this study, we did not find any *SbxSc* hybrid species of bovine schistosome, nor any hybrid between *ShxSb* or *ShxSc*, as recently highlighted in Cameroon [12]. However, *SbxSc* hybrids have been reported in cattle in Senegal, Niger, and Mali [6, 36, 44] and sheep and goats, in Niger [7, 8]. The absence of hybrid parasites could be explained by the fact that the transmission sites we sampled were characterized by a high prevalence of either *Sb* or *Sc* or by a low rate of co-infected cattle. In the latter case, interspecific encounters are infrequent and hybridization unlikely. In addition, we collected adult parasites without identifying whether we had inter-specific pairs. Genotyping the eggs might have enabled us to identify first-generation hybrid eggs, as observed in Benin [38].

Regardless of the method used (Fst, PCA or Structure), there is strong structuring between *Sb* and *Sc* species, and weak structuring between slaughterhouses within the same species. Concerning the genetic difference between species, most published studies are based on sequence comparisons, and in particular, involve sequencing the cytochrome oxidase gene [18]. In the latter study, the authors measured a genetic difference of 6.1% between *Sb* and *Sc*, in Senegal. Based on microsatellite markers, we measured a genetic differentiation of 4.2% between these two species. Concerning the difference between populations within the same species, we measured an Fst of 0.43% between the CBS and SS abattoirs for *Sb*. These two abattoirs are about 18 km apart. This result is in agreement with the weak genetic structuring of *Sb* observed in Cameroon [12] and Côte d’Ivoire [15]. With only two sites, it is difficult to provide more general results on the genetic structure of this parasite species in Mali. As Mali is the leading livestock-producing country in the West African Economic and Monetary Union, it is natural for livestock to move across the country and even across borders in search of pasture, which encourages the spread of parasites. During this transhumance, it is often possible for herders to sell animals along the way [2]. Moreover, cattle trade between countries is universally present in West Africa. It is possible that some of the cattle sampled in this study came from countries bordering Mali. As Bamako is the country’s capital, it receives cattle from all areas, including Burkina-Faso, Niger, and even Senegal. All of these movements may be at the root of the weak structuring within the various species. The strong presence of *Sc* in the Kayes slaughterhouse is easily explained by its proximity to Mauritania and Senegal, where this species has already been reported [17, 44]. Being able to use the same panel of microsatellite markers on *Sh*, *Sb,* and *Sc* is an opportunity to carry out more studies on animal parasites and to infer their possible role in schistosome transmission to humans. For instance, our study shows that the heterozygosity rate is higher for *Sb* than for *Sc.* Variations in heterozygosity have already been observed in previous studies between *Sb* and *Sh* in Cameroon [12] and in Côte d’Ivoire [15].

Our work has a number of limitations. Because the worms were grouped by animal and by organ, we were unable to isolate the pairs directly in the abattoir and we were unable to obtain the genotypes/species of each pair. It is also incredibly difficult to collect parasites in abattoirs. The time allowed per animal is always very limited so as not to disrupt the slaughter line. In addition, we limited our study to cattle. It would have been interesting to also inspect sheep and goats.

Animal schistosomiasis is prevalent in cattle in the Kayes and Bamako abattoirs, with the highest prevalence found in Kayes. Our study focused on the genetic profile and diversity of schistosome adults collected from livestock in Mali. Two pure species were found (*Sb* and *Sc*). Parasites were well structured according to species, with *Sb* and *Sc* dominating in Bamako and Kayes, respectively. Further studies on the genome of animal schistosomes on a large scale and the potential impacts of hybrid strains on animals are required. Additional studies are also needed to expand our understanding in this field by increasing the sample size and by extending the surveys to other slaughterhouses and to farm livestock in Mali. In view of our findings, a strategic control program for cattle trematodes is warranted. An important component of such a control program is to water cattle in troughs rather than to let them water freely from rivers and ponds, in order to reduce fecal contamination.

**Abbreviations**
*Sb*
: *Schistosoma bovis*
*Sc*
: *Schistosoma curassoni*
ITS: Internal transcribed spacer
Cox1: Cytochrome oxidase subunit 1 gene
RD-PCR: Rapid diagnostic multiplex PCR
T-ARMS-PCR: Tetra-amplification refractory mutation system PCR
FTA: Flinders Technology Associates
DNA: Deoxyribonucleic acid
UEMOA: Union Économique et Monétaire Ouest Africaine
ECOWAS: Economic Community of West African States
PRODEVIM: Programme de Développement à l’Exportation de la Viande du Mali
PCA: Principal Component Analysis
CBS: Central Bamako Slaughterhouse
SS: Sabalibougou Slaughterhouses
KS: Kayes Slaughterhouse
He: Expected heterozygosity
Ho: Observed heterozygosity
A: Allele
Ar: Allelic richness
Fis: Inbreeding coefficient
*B*
: *Bulinus*

## Data Availability

All data generated or analyzed during this study are included in this published article.
